# Increased tolerance to salt stress in OPDA-deficient rice *ALLENE OXIDE CYCLASE* mutants is linked to an increased ROS-scavenging activity

**DOI:** 10.1093/jxb/erv142

**Published:** 2015-04-06

**Authors:** Mohamed Hazman, Bettina Hause, Elisabeth Eiche, Peter Nick, Michael Riemann

**Affiliations:** ^1^Botanical Institute, Molecular Cell Biology, Karlsruhe Institute of Technology, Karlsruhe, Germany; ^2^Agricultural Genetic Engineering Research Institute (AGERI), Agricultural Research Centre (ARC), Giza, Egypt; ^3^Cell and Metabolic Biology, Leibniz Institute of Plant Biochemistry (IPB), Halle, Germany; ^4^Institute of Applied Geosciences, Karlsruhe Institute of Technology (KIT), Karlsruhe, Germany

**Keywords:** ALLENE OXIDE CYCLASE (AOC), jasmonate, *Oryza sativa*, oxidative stress, 12-oxophytodienoic acid (12-OPDA), reactive oxygen species (ROS), salinity.

## Abstract

Salinity stress represents a global constraint for rice, the most important staple food worldwide. Therefore the role of the central stress signal jasmonate for the salt response was analysed in rice comparing the responses to salt stress for two jasmonic acid (JA) biosynthesis rice mutants (*cpm2* and *hebiba*) impaired in the function of ALLENE OXIDE CYCLASE (AOC) and their wild type. The *aoc* mutants were less sensitive to salt stress. Interestingly, both mutants accumulated smaller amounts of Na^+^ ions in their leaves, and showed better scavenging of reactive oxygen species (ROS) under salt stress. Leaves of the wild type and JA mutants accumulated similar levels of abscisic acid (ABA) under stress conditions, and the levels of JA and its amino acid conjugate, JA–isoleucine (JA-Ile), showed only subtle alterations in the wild type. In contrast, the wild type responded to salt stress by strong induction of the JA precursor 12-oxophytodienoic acid (OPDA), which was not observed in the mutants. Transcript levels of representative salinity-induced genes were induced less in the JA mutants. The absence of 12-OPDA in the mutants correlated not only with a generally increased ROS-scavenging activity, but also with the higher activity of specific enzymes in the antioxidative pathway, such as glutathione *S*-transferase, and fewer symptoms of damage as, for example, indicated by lower levels of malondialdehyde. The data are interpreted in a model where the absence of OPDA enhanced the antioxidative power in mutant leaves.

## Highlight

The lack of the jasmonic acid precursor 12-oxophytodienoic acid in rice led to increased salt tolerance, which is correlated with an increased ROS-scavenging activity in stress conditions.

## Introduction

Soil salinization is a global threat that causes a huge reduction of agricultural yield worldwide. More than 20% of all irrigated land on earth is affected by salinization. Rice (*Oryza sativa*), as one of the world’s most important cereal crops, provides the primary source of food and calories for about half of mankind (Khush, 2005). Rice as a so-called glycophyte is very sensitive to salinity stress especially at the seedling stage, with height, root length, emergence of new roots, and dry matter affected significantly by salinity (Pearson *et al.*, 1966; Akbar and Yabuno, 1974). Salinity stress is generally defined as the presence of excessive amounts of soluble salt that hinder or negatively affect the functions needed for normal plant growth and development. Salt stress is comprised of two harmful effects: osmotic stress leading to reduced water uptake, and ionic stress caused by the toxicity of specific ions (mainly Na^+^ and Cl^–^). Ionic stress leads to unrestrained overproduction of ROS (reactive oxygen species), such as superoxide radicals (O_2_·), hydrogen peroxide (H_2_O_2_), and hydroxyl radicals (OH·^–^). These reactive molecules accumulate to toxic levels and trigger oxidative damage in the cells and organelles by destroying membranes, proteins, enzymes, and nucleic acids (Abogadallah, 2010; Sharma *et al.*, 2012; Ismail *et al.*, 2014*a*, *b*).

The adaptive response of salt-stressed plants is controlled by chemical signals that will compensate adjustment of growth and development in response to such unfavourable conditions. It should be noted that some of these signals play a dual role—if controlled in space and time, they can act as signals triggering adaptation, if developing unconstrained, they accompany stress-related damage (for a review, see Ismail *et al.*, 2014*b*). Central players among these stress signals are jasmonic acid (JA), its biologically active precursor 12-oxophytodenoic acid (OPDA), and its derivatives such as methyl jasmonate (MeJA) or the amino acid-conjugated jasmonate, JA–isoleucine (JA-Ile), in the following collectively termed as jasmonates (JAs). JAs have been reported to accumulate in response to salinity stress (tomato, Pedranzani *et al.*, 2003; rice, Moons *et al.*, 1997). Whether this accumulation is a signal triggering adaptation or just a by-product or consequence of adaptation is not very clear. However, the fact that a salt-tolerant cultivar of rice shows higher endogenous JA contents as compared with a salt-sensitive cultivar, as well as the observation that exogenous MeJA can reduce the uptake of sodium in this salt-tolerant cultivar (Kang *et al.*, 2005), indicates a function for JAs in salt adaptation. Overexpression of a wheat *AOC* (ALLENE OXIDE CYCLASE) gene in wheat and *Arabidopsis* resulted in an improved salt tolerance of these species (Zhao *et al.*, 2014). However, it is not possible to draw a general connection between high levels of JA and adaptation; during a comparison of two grapevine cell lines differing in their salinity tolerance, the accumulation of JA and JA-Ile was more pronounced in the sensitive *Vitis riparia* rather than in the salt-tolerant *Vitis rupestris* (Ismail *et al.*, 2014*a*). These discrepancies underscore that it is not the presence or absence of JAs that decides the salinity response, but rather the right timing and control (for a review, see Ismail *et al.*, 2014*b*). The complexity in the relationship between JAs and salinity adaptation is further accentuated by the recent finding that the precursor OPDA (but not JA itself) was significantly induced in drought-stressed *Arabidopsis* leaves (Savchenko *et al.*, 2014). Moreover, in rice roots, JA biosynthesis was reported to be strongly induced by drought stress, but only marginally by salt stress, indicating that the two components of salinity stress might differ in their transduction events (Takeuchi *et al.*, 2011).

The complexity extends into the signalling triggered by JAs, because this signalling has been found to interact with the signalling triggered by other plant hormones known to be involved in the adaptation to salt stress such as abscisic acid (ABA). These interactions, often referred to as ‘hormonal cross-talk’, need more investigations, especially in crops of economic importance. For instance, MYC2, a transcription factor conveying the stimulation of transcription by JA, also acts downstream of ABA, providing a mechanism for co-regulation of the respective target genes (Kazan and Manners, 2012). The addition of exogenous MeJA caused an elevation in the endogenous level of ABA in rice (Seo *et al.*, 2001), indicating that ABA synthesis is modulated by JA signalling. In *Arabidopsis thaliana*, JA induces expression of genes encoding ABA receptor proteins, thereby contributing to a maintenance of the balance between growth and defence (for a review, see Wasternack and Hause, 2013). However, the interactions can also be antagonistic, as found for the regulation of salt-stress-related gene expression by JAs and ABA in rice and *Arabidopsis* (Moons *et al.*, 1997; Anderson *et al.*, 2004). In addition to MYC2, JA and ABA signalling interact at the level of the JASMONATE ZIM-DOMAIN (JAZ) gene family, acting as repressors for JA signalling (Staswick, 2008). These two, only partially elucidated mechanisms provide a tight regulatory interaction between JA- and ABA-mediated signalling during the response to salt stress (for a review, see Kumar *et al.*, 2013).

As one of the most salt-sensitive glycophytes, rice has very limited strategies to deal effectively with salt stress and therefore does not grow well on saline soil (Galvan-Ampudia and Testerink, 2011). Therefore, lowering the amount of sodium loaded into the transpiration stream is vital to circumvent damage of photosynthetic tissues. Salinity and osmotic stress induce, either directly or indirectly via hormonal regulation, stomatal closure, which reduces evaporation and overall water transport (Horie *et al.*, 2012). At micromolar concentrations, ABA causes closure of stomata, but the effect of ABA on the stomatal aperture is dependent on the intercellular CO_2_ concentration and on the presence of the signal substance nitric oxide (NO) (Heldt and Heldt, 2005). Due to the close interaction between JA and ABA signalling, it is to be expected that JAs also play a role in stomatal aperture. However, it should be noted that due to its negative impact on photosynthesis, stomatal closure can only provide short-term protection due to reduced transpiration, but cannot be a mechanism for long-term adaptation to salinity stress.

To show the functionality of JAs for adaptation, in previous studies exogenous MeJA was added (Kang *et al.*, 2005) and/or fluctuations in the levels of endogenous JAs were measured (Moons *et al.*, 1997; Pedranzani *et al.*, 2007). However, these fluctuations could be by-products of adaptation rather than their cause, and exogenous MeJA is present constitutively and therefore is not a good equivalent for the tightly controlled, transient accumulation of JAs that is characteristic for successful adaptation. To address the functionality of JAs for adaptation, it is necessary to create a condition in which the accumulation of endogenous JAs is blocked. This became possible for the rice model by mutants impaired in JA biosynthesis (Riemann *et al.*, 2003). The mutant *hebiba* is deficient in JA, and this phenotype could be attributed to the locus for AOC, a synthetic enzyme driving the formation of the JA precursor OPDA (Riemann *et al.*, 2013). A second mutant, *coleoptile photomorphogenesis 2* (*cpm2*), shows a similar phenotype and could be shown to be allelic to *hebiba*. Using these two mutants of JA biosynthesis in comparison with their wild-type background, the role of JA for salinity adaptation was analysed in an integrative approach considering the response of growth, ion uptake, and redistribution, ROS, antioxidants, accumulation of phytohormones, and the expression patterns of specific response genes. A model was arrived at where the absence of OPDA accumulation in the mutants enables a better capability to ward off oxidative stress.

## Materials and methods

### Plant materials, growth, and stress conditions

In this study, *Oryza sativa* L. *ssp. japonica* cv. Nihonmasari was used as the wild type. The two mutant lines *cpm2* and *hebiba* were generated in the same cultivar (Riemann *et al.*, 2013). The caryopses were dehusked and surface sterilized by incubating the seeds in 70% ethanol for 1min then washed briefly twice with double-distilled water. Subsequently, the seeds were incubated in a sodium hypochlorite solution containing ~5% of active chlorine for 30min followed by five washing steps in sterilized double-distilled water. The seeds were sown on 0.5% phytoagar medium (Duchefa, The Netherlands) and incubated for 10–12 d in a culture room (at 25 °C, continuous light of 120 μmol m^–2^s^–1^). After 10–12 d, well-grown seedlings were transferred to custom-made sterilized floating racks and moved to a glass container containing double-distilled water as control or a solution containing NaCl to cause salt stress. The shoots of control and stressed plants were harvested, frozen in liquid nitrogen, and then stored in –80 °C to be used for enzymatic or non-enzymatic antioxidants, or for gene expression analysis.

### Analysis of root elongation

Root elongation was evaluated as the mean of the seminal root length of seedlings raised in darkness (25 °C, 7 d). The seeds were surface sterilized as described above, and sown on 0.5% phytoagar medium with different concentrations of NaCl (0, 7.8, 15.6, 31.3, 62.3, 125, and 250mM). The seedlings were scanned and the root length was measured using Image J (http://imagej.nih.gov/ij/, last accessed 22 January 2015).

### Measurement of sodium ion content

Dry cells of each biological replicate were transferred into digestion tubes (Gerhardt, UK), supplemented with 5ml of concentrated nitric acid (HNO_3_), and then incubated for at least 24h at room temperature with vortexing at 6h and 24h. Samples were placed on a water bath at 100 °C for 2h. After cooling, the final volume of each sample was adjusted to 10ml with distilled water and vortexed. Contents of sodium ions were measured by flame atomic absorption spectrometry (AAnalyst200, Perkin Elmer) in an air acetylene flame (Institute of Applied Geosciences, Aquatic Geochemistry, Karlsruhe Institute of Technology). Blank samples were prepared by adding 5ml of concentrated nitric acid to an empty digestion vessel and processed as described above.

### Determination of lipid peroxidation and aqueous peroxide levels

Lipid peroxidation of shoots was estimated by the level of malondialdehyde (MDA) using the thiobarbituric acid (TBA) method as described by Heath and Packer (1968). Briefly, 500mg of rice shoots were homogenized using a mortar and pestle in 1ml of 0.1% trichloroacetic acid (TCA, w/v). The homogenate was then centrifuged at 10 000 *g* for 20min, and 0.5ml of the supernatant was added to 1ml of 0.5% TBA in 20% TCA. This mixture was then heated in a boiling water bath for 1h. The reaction was stopped by transferring the tubes to an ice bath for 10min, and then the tubes were centrifuged for 10min at 10 000 *g*. The absorbance of the supernatant was recorded at 532nm and 600nm. The value of the non-specific absorption at 600nm was subtracted. The amount of MDA–TBA complex (red pigment) was calculated from the extinction coefficient 155mM^–1^ cm^–1^.

Steady-state levels of H_2_O_2_ in the shoots of control and salt-stressed rice seedlings were measured using the FOX-1 method (ferrous oxidation with Xylenol Orange) (Wolf, 1994) with some modifications. Leaves (70mg fresh weight) were perfectly ground in 5ml of 5% TCA containing 100 μg of active charcoal. The mixture was filtered using No. 1 filter paper (Whatman), and then a measured volume of the filtrate was incubated with FOX-1 reagent (100 μM Xylenol Orange, 250 μM ammonium sulphate, 100mM sorbitol, and 25mM H_2_SO_4_) for 30min. The absorbance was recorded at 560nm. The values of aqueous peroxide were referred to as micromoles H_2_O_2_ using a standard curve.

### Estimation of soluble proline

Accumulation of free proline was monitored according to Bates *et al.* (1973). Briefly, 200mg of fresh tissue of leaves were homogenized using a mortar and pestle containing a small amount of quartz sand. The homogenate was filtered through filter paper No. 1 (Whatman). The filtrate was centrifuged (10 000 *g*, 10min) at room temperature. A 1ml aliquot of the supernatant was treated with 2ml of reaction buffer (1ml of glacial acetic acid and 1ml of ninhydrin reagent), and mixed well. The reaction was boiled in a water bath for 1h, and then cooled to room temperature gradually. The absorbance was recorded at 520nm. Soluble proline content was expressed as μmol proline per g fresh weight according to a standard curve.

### Free radical-scavenging activity (DPPH-scavenging activity)

In order to measure the antioxidant ability of the rice shoot extract, 2, 2-diphenyl-1-picrylhydrazyl (DPPH), a stable free radical, was used according to Goffman and Bergman (2004). A crude extract (100 μl) of rice leaves at different concentrations (37–370 μg extract ml^–1^ reaction) was added to 900 μl of freshly prepared DPPH methanolic solution (80 ppm). The reduction in the absorbance (515nm) of the methanolic solution of DPPH was monitored. The percentage DPPH inhibition was calculated using the following formula: (Ab_cont_–Ab_sample_)/Ab_cont_×100, with the Ab_cont_ absorbance value at 515nm. The IC_50_ value for each sample was calculated to determine the amount in micrograms of extract sufficient to scavenge 50% or half of the DPPH radical substance. Butylated hydroxyanisole (BHA), as a very efficient antioxidant, was used as an internal control.

### Antioxidant enzyme activity measurement

For estimating the activity of catalase (CAT), peroxidase (POD), glutathione reductase (GR), glutathione *S*-transferase (GST), and superoxide dismutase (SOD), leaves of the treated seedlings were homogenized in 1ml of ice cold extraction buffer according to Venisse *et al.* (2001). For ascorbate peroxidase (APX), the same procedure was followed but using the extraction buffer of Nakano and Asada (1981). SOD (EC 1.5.1.1) activity was assayed by monitoring the inhibition of the photochemical reduction of nitroblue tetrazolium at 560nm (Beauchamp and Fridovich, 1971). Total protein content was estimated according to Bradford *et al.* (1976).

### RNA extraction and quantitative real-time PCR

Total RNA was isolated from the shoots of control and salinity-stressed plants (100mM NaCl, 24h and 72h) using the InnuPrep plant RNA kit (Analytika Jena RNA kit) according to the manufacturer’s instructions. cDNA synthesis was performed with a Dynamo cDNA synthesis kit (Finnzymes, Finland) using total RNA as a template. Real-time PCR was done with a SYBR green dye protocol using an Opticon 2 system (Biorad, USA). The primer sequences for the genes of interest are listed in Supplementary Table S1 available at *JXB* online.

### Endogenous level of ABA, OPDA, JA, and JA-Ile

OPDA, JA, JA-Ile, and ABA were quantified simultaneously using a standardized ultraperformance liquid chromatography–tandem mass spectrometry (UPLC-MS/MS)-based method according to Balcke *et al.* (2012) using [^2^H_5_]OPDA, [^2^H_6_]JA, [^2^H_2_]JA-ile, and [^2^H_6_]ABA as internal standards.

### Chlorophyll content

Total chlorophyll, chlorophyll *a*, and chlorophyll *b* contents were determined based on the method of Arnon (1949). A 100mg aliquot of leaves was homogenized with acetone. The extract was filtered through Whatman No. 1. filter paper and washed 2–3 times with 80% acetone. The final volume of the extract was made up to 25ml. Chlorophyll contents were calculated based on the absorbance measured at 645, 652, and 663nm, respectively.

### Preparation of plant extracts for the determination of specific ROS-scavenging activities

Rice leaves were collected and washed gently several times with Millipore water. The washed leaves were ground in 5ml of warm Millipore water (80 °C). Subsequently the homogenate was mixed with 50ml of Millipore water, stirred for 3h, and filtered through filter paper No. 2 (Whatman). This process was repeated once and the combined filtrate was freeze-dried. The yellowish solid crude extracts were kept at –20 °C to be used for testing scavenging activities of O_2_·, H_2_O_2_, and OH·^–^ as described below.

### Superoxide anion-scavenging activity

Measurement of superoxide-scavenging activity was done based on the method described by Zhishen *et al.* (1999) with slight modifications. All solutions were prepared in 0.05M phosphate buffer (pH 7.8). The reaction was carried out in light for 40min in a total volume of 3ml. Crude extracts were dissolved in phosphate buffer to obtain aqueous extracts in the final concentrations of 25, 50, 100, 150, and 250 μg per reaction, respectively. Absorbance was measured at 560nm. Low absorbance of the reaction mixture indicates increased O_2_·-scavenging activity. The percentage inhibition of O_2_· generation was calculated by using the following formula:

$$\text{O2}\cdot^{–}-\text{scavenging effect }\left(\%\right)=\left[\left({\text{A}}_{0}–{\text{A}}_{1}\right)/{\text{A}}_{0}\right]\times \text{1}00$$

A_0_ is the absorbance of the control with water instead of aqueous extract, while A_1_ is the absorbance of sample or standard, respectively. The IC_50_ value is the concentration of sample in micrograms of aqueous extract per reaction required for scavenging 50% of O_2_· in the reaction mixture. It was calculated based on a regression curve.

### Hydrogen peroxide-scavenging assay

H_2_O_2_-scavenging activity of the extract was determined by the method of Ruch *et al.* (1989). Different amounts (60, 120, 240, 300, and 420 μg) of the extract were dissolved in 3.4ml of 0.1M phosphate buffer (pH 7.4), and mixed with 600 μl of a 43mM solution of H_2_O_2_. The absorbance value of the reaction mixture was recorded at 230nm and butylhydroxytoluol (BHT) was used as a standard. The percentage scavenging of H_2_O_2_ was calculated as follows

$${\text{Inhibition of H}}_{\text{2}}{\text{O}}_{2}\left(\%\right)=\text{1}-{\text{A}}_{\text{Sample 23}0\text{nm}}/{\text{A}}_{\text{Control 23}0\text{nm}}\times \text{1}00$$

The IC_50_ value is the concentration of sample in micrograms of aqueous extract per reaction required for scavenging of 50% of H_2_O_2_ in the reaction mixture, and it was calculated based on a regression curve.

### Hydroxyl radical-scavenging assay

OH·-scavenging activity was measured according to the method of Kunchandy and Rao (1990). The reaction mixture contained 0.8ml of phosphate buffer solution (50mM, pH 7.4), 0.2ml of a sample of different concentrations (25, 50, 100, 150, and 250 μg of aqueous extract per reaction mixture), 0.2ml of EDTA (1.04mM), 0.2ml of FeCl_3_ (1mM), and 0.2ml of 2-deoxyribose (60mM). The mixtures were kept in a water bath at 37 °C and the reaction was started by adding 0.2ml of ascorbic acid (2mM) and 0.2ml of H_2_O_2_ (10mM). After incubation at 37 °C for 1h, 2ml of cold TBA (10 mgml^–1^) was added to the reaction mixture followed by 2ml of HCl (25%). The mixture was heated at 100 °C for 15min and then cooled down with water. The absorbance of the solution was measured at 532nm. The scavenging percentage was calculated according to the following formula:

$$\text{Scavenging percentage }\%=\left[{\text{A}}_{0}-\left({\text{A}}_{1}-{\text{A}}_{2}\right)\right]\times \text{1}00/{\text{A}}_{0}$$

where A_0_ is the absorbance of the control without a sample. A_1_ is the absorbance after adding the sample and deoxyribose, and A_2_ is the absorbance of the sample without deoxyribose. The IC_50_ value is the concentration of sample in micrograms of total aqueous extract per reaction required for the scavenging of 50% of OH· in the reaction mixture. It is calculated based on the regression curve.

## Results

### JA-deficient mutants are less sensitive to salinity stress

**Fig. 1.**
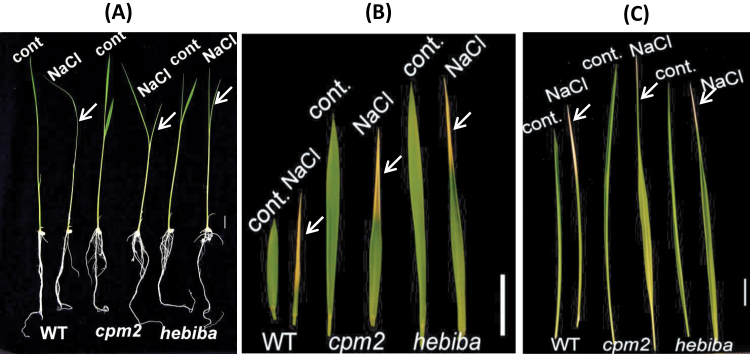
Effects of salt stress on the phenotype of the wild type (WT) and JA biosynthesis mutants. (A) Ten-day-old rice seedlings were subjected to salt stress at a high concentration (285mM NaCl) for 24h. (B) The second leaf of 10-day-old rice seedlings which were subjected to a 100mM NaCl solution for 3 d. (C) The third leaf of 10-day-old rice seedlings which were subjected to a 100mM NaCl solution for 3 d. Arrows indicate the salt-treated leaves. Scale bar=10mm.

Rice seedlings (age 10 d) were subjected to salt stress either at a very high concentration for short-term experiments (285mM NaCl for 24h), or at a high concentration for long-term experiments (100mM for 72h, subsequently referred to as mid-term exposure). For both stress treatments, leaves of *hebiba* and *cpm2* appeared to be less sensitive with respect to wilting and necrosis (Fig. 1). In the short-term exposure at high concentrations, the wild-type leaves were entirely rolled as a response to salt stress, whereas the mutant leaves remained unfolded (Fig. 1A). Moreover, the second leaf of the wild type was almost entirely necrotic after mid-term salt exposure, whereas the second leaves of both mutants showed necrosis only at the tip (Fig. 1B). For the same treatment, the third leaf of the wild type was entirely rolled and almost half of the leaf was necrotic, starting from the tip (Fig. 1C). In contrast, the third leaves of both *hebiba* and *cpm2* were only marginally necrotic, at the tip, and approximately two-thirds of the leaf remained unfolded. In addition, they were also able to maintain higher chlorophyll levels under salt stress (Supplementary Fig. S1 at *JXB* online). Hence, these mutants, which are deficient in the phytohormone JA, displayed a clearly reduced salt sensitivity in their phenotype. It is noteworthy that mutants develop longer leaves due to deficiency in JA, which has been described for *hebiba* previously (Riemann *et al.*, 2003).

### Effect of salt stress on root elongation

**Fig. 2.**
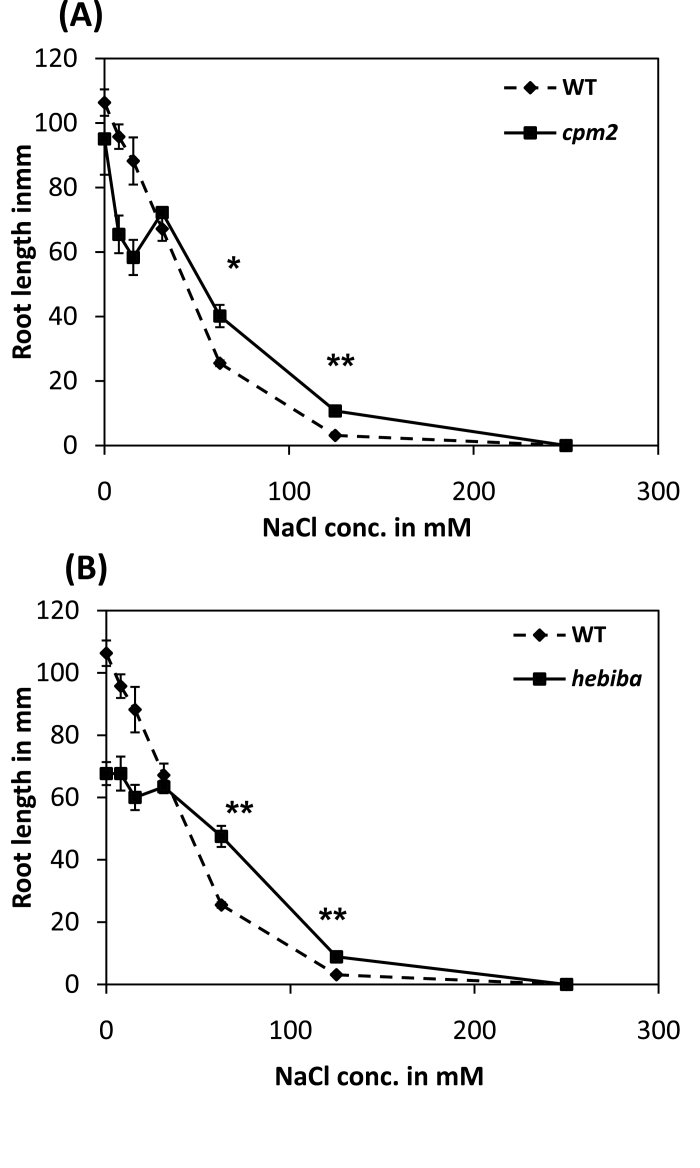
Root length of the wild type (WT) and JA biosynthesis mutants grown in medium containing different concentrations of NaCl. (A) Comparison of the root length of etiolated WT and *cpm2* seedlings grown on medium containing 0–250mM NaCl. (B) Comparison of the root length of etiolated WT and *hebiba* seedlings grown on medium containing 0–250mM NaCl. *n*=70–100 seedlings. *Significant difference at *P*<0.05; **significant difference at *P*<0.01 in a Student’s *t*-test.

In order to compare the effect of salinity on growth in the wild type and mutants, the length of seminal roots was examined after 7 d in complete darkness when caryopses were germinated on medium containing various amounts of NaCl. Roots of *hebiba* and *cpm2* were shorter under these conditions when grown in the absence of supplemented NaCl or at concentrations <12mM (Fig. 2), indicating a function for AOC in seminal root elongation. When salt concentrations were raised to 62.5mM and 125mM, both wild-type and mutant roots decreased in length. However, this decline was less pronounced in the mutants, such that, under these conditions, mutant roots were significantly longer than those of the wild type (Fig. 2). Neither the wild type nor the JA biosynthesis mutants was able to germinate on medium containing 250mM NaCl.

### JA biosynthesis mutants accumulate less Na^+^ ions in shoots, but not in roots

**Fig. 3.**
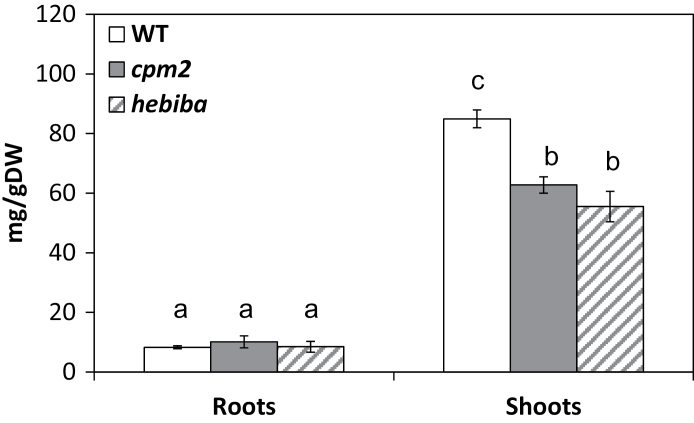
Content of sodium ions in roots and shoots of the wild type (WT) and JA biosynthesis mutants. Ten-day-old rice WT (white bars), *cpm2* (grey bars), and *hebiba* (striped bars) seedlings were stressed in aqueous NaCl (100mM) solution for 3 d in roots and in shoots. Values represent the mean of at least three independent experiments ±SE. Significant differences amongst different treatments or genotypes are indicated by different letters, according to Tukey’s Honestly Significant Difference (HSD) test (*P*<0.05).

The degree of stress will depend on the uptake and translocation of NaCl. Therefore, the content of sodium ions was examined in roots and shoots of seedlings grown for 3 d on 100mM NaCl. The shoots of the mutants accumulated significantly (by 25–30%) less sodium ions compared with the wild type. However, there was no significant difference between the mutants and the wild type in sodium contents in the roots (Fig. 3). Hence, the weaker stress symptoms observed in mutant leaves correlate with a lower level of sodium ions in the tissue.

### Enhanced antioxidant activity in JA-deficient mutants

**Fig. 4.**
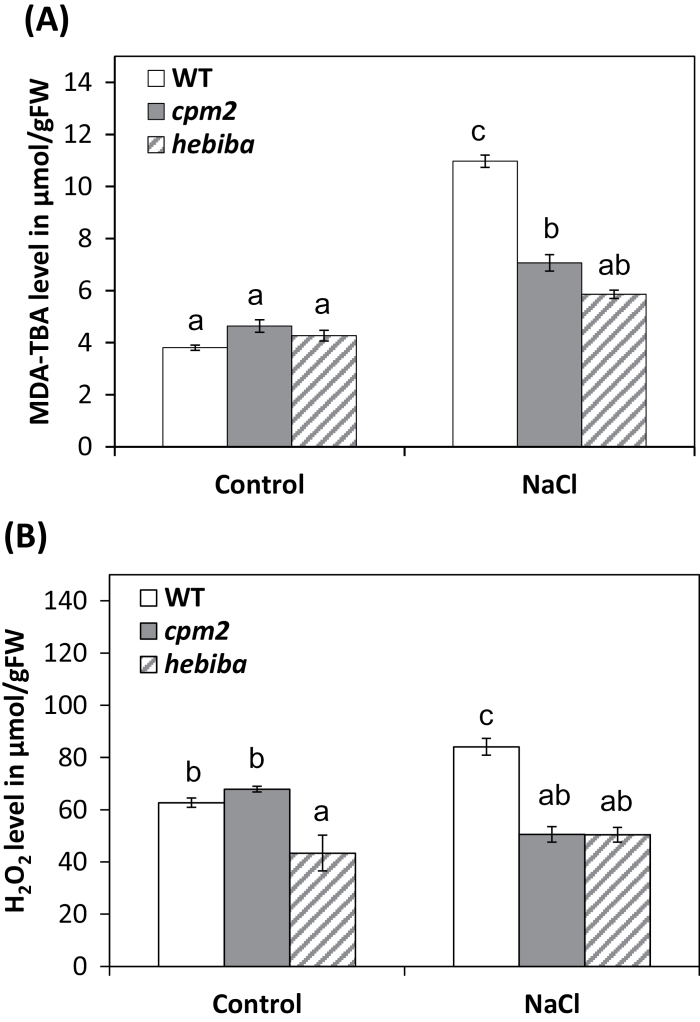
Lipid peroxidation and hydrogen peroxide levels in the wild type (WT) and JA biosynthesis mutants under salinity stress. (A) The level of malondialdehyde (MDA) was estimated in the WT and JA biosynthesis mutants in shoots of control and salt-stressed seedlings. (B) Levels of aqueous peroxide in the WT and JA biosynthesis mutants in shoots of control and salt-stressed seedlings. Values represent the mean of at least three independent experiments ±SE. Results for the WT, *cpm2*, and *hebiba* are indicated by white, grey, and striped bars, respectively. Significant differences amongst different treatments or genotypes are indicated by different letters, according to Tukey’s Honestly Significant Difference (HSD) test (*P*<0.05).

Oxidative damage is a manifestation of stress susceptibility. Therefore oxidative events and the antioxidative system of wild-type and JA biosynthesis mutant plants were analysed under salinity stress. First, the level of lipid peroxidation, as a readout for oxidative degradation of the membrane, was estimated by measuring the product MDA. Generally, the level of MDA was increased in both the wild type and JA mutants in response to salt stress. Nevertheless, the salinity-induced level of MDA in wild-type shoots was ~50% higher compared with *cpm2* and almost twice that observed in *hebiba* (Fig. 4A). To understand the reduced lipid peroxidation in the mutants, the levels of H_2_O_2_ in the shoot were determined using Xylenol Orange (Fig. 4B). In the unchallenged controls, the content of H_2_O_2_ was similar for the wild type and *cpm2*, whereas *hebiba* displayed significantly lower (30%) values. In response to salt stress, the wild type accumulated ~ 60% more H_2_O_2_ compared with the mutants. Thus, the pattern for H_2_O_2_ paralleled the level of lipid peroxidation reported by MDA.

**Fig. 5.**
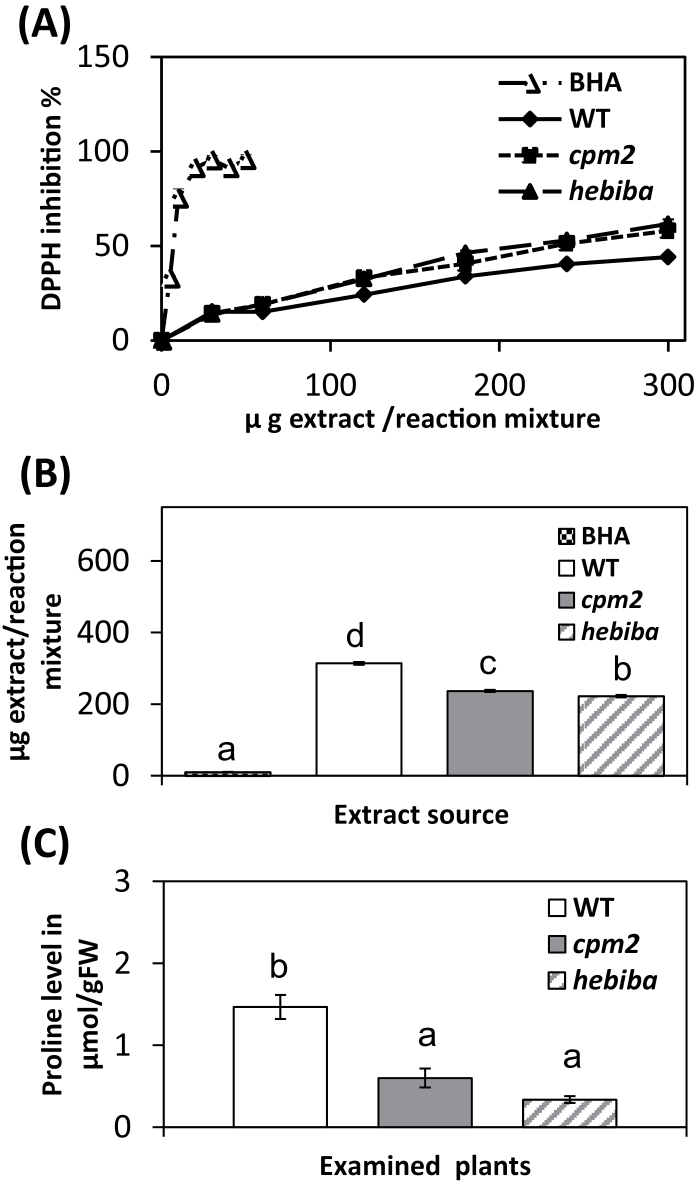
Free radical-scavenging ability and level of soluble proline in the wild type (WT) and JA biosynthesis mutants. (A) DPPH free radical-scavenging activity of standard butylated hydroxyanisole (BHA) and methanolic extract of WT and JA biosynthesis mutants under salinity stress of 100mM NaCl for 3 d. (B) IC_50_ values were calculated from (A) depending on regression analysis. (C) Soluble proline in the WT, *cpm2*, and *hebiba* under salt stress. Proline was not detectable in control samples of the same genotypes. Values represent the mean of at least three independent experiments ±SE. Results for the WT, *cpm2*, and *hebiba* are indicated by white, grey, and striped bars, respectively. The dotted symbols in (A) and (B) represent the measurement for BHA. Significant differences amongst different treatments or genotypes are indicated by different letters, according to Tukey’s Honestly Significant Difference (HSD) test (*P*<0.05).

In order to test the ability of the plants to detoxify the ROS, the total antioxidative ability was estimated, by quantifying scavenging of the stable radical DPPH by methanolic leaf extracts. The methanolic extract of all three genotypes succeeded in scavenging the stable radical DPPH in a dose-dependent manner, however, with different efficiency (Fig. 5A). To quantify these differences, the concentrations required to reach 50% inhibition were determined and these IC_50_ values were plotted as a measure of scavenging activity (Fig. 5B). Compared with the wild type, leaf extracts from the mutants were more efficient, with IC_50_ values being reduced by about a third, whereby *hebiba* was most efficient. As a positive control, the powerful scavenger BHA was used. The very low IC_50_ value of 8.23 μg ml^–1^ confirms that the assay system was working properly. Thus, the reduced accumulation of MDA in the JA biosynthesis mutants correlates with an elevated activity of antioxidants. To understand this improved ROS detoxification in more detail, additional assays were used that can discriminate between the different species of active oxygen, namely O_2_·^–^, H_2_O_2_, and OH·^–^, testing extracts from both control and salt-stressed plants. As shown in Supplementary Fig. S2 at *JXB* online, aqueous leaf extracts from both *cpm2* and *hebiba* scavenged O_2_· better than the wild type, with IC_50_ values that were 30% (*cpm2*) to 40% (*hebiba*) lower than in the wild type. In contrast, there was no difference between the wild type and JA biosynthesis mutants in the detoxification of H_2_O_2_ and OH· (Supplementary Figs S3, S4). In addition to scavenging superoxide, plants under salt stress accumulate soluble proline as a protection against ion-dependent protein degradation. The level of soluble proline was therefore estimated. The control samples of both the wild type and the mutants did not contain a detectable amount of soluble proline (Fig. 5C). In all the genotypes tested, exposure to salinity led to a considerable increase in proline content. However, the estimated soluble proline amount in the shoots of the mutants was significantly lower than in those of the wild type (by 30% in *cpm2*, and even by 60% in *hebiba*).

### Effect of salinity on the activity of antioxidative enzymes

**Fig. 6.**
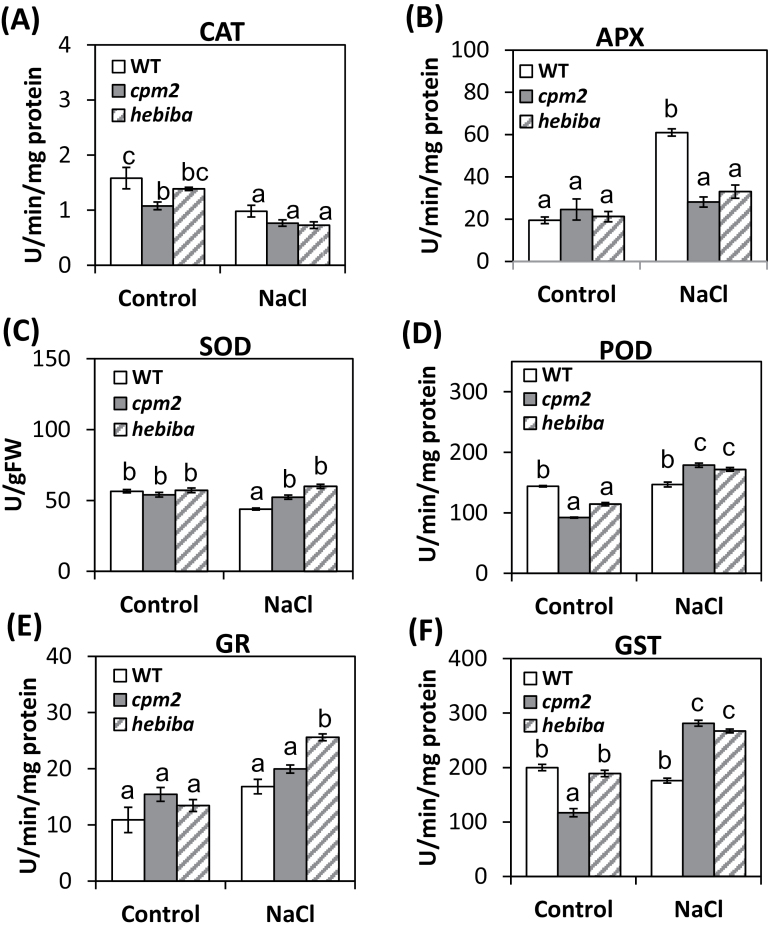
Profiles of antioxidative enzymes in both the wild type (WT) and JA biosynthesis mutants under salinity stress. Activity of (A) catalase (CAT), (B) ascorbate peroxidase (APX), (C) superoxide dismutase (SOD), (D) peroxidase (POD), (E) glutathione reductase (GR), and (F) glutathione *S*-transferase (GST). Values represent the mean of at least three independent experiments ±SE. Results for the WT, *cpm2*, and *hebiba* are indicated by white, grey, and striped bars, respectively. Significant differences amongst different treatments or genotypes are indicated by different letters, according to Tukey’s Honestly Significant Difference (HSD) test (*P*<0.05).

To understand in more detail by what mechanisms the mutants achieve their improved antioxidant homeostasis, the activities of the enzymatic scavengers CAT, APX, SOD, POD, GR, and GST were determined. Activities were estimated in the shoots of the wild type and JA biosynthesis mutants following treatment with 100mM NaCl over 72h. The activity of CAT was diminished in the shoots of the wild type, *cpm2*, and *hebiba* after salt stress (Fig. 6A), and the level of activity under salt stress was not significantly different between the wild type and mutants. In contrast, APX activity was increased in response to salinity stress by almost 3-fold in the wild type but was not significantly increased in the mutants (Fig. 6B). The activity of SOD decreased slightly under salt stress in the wild type, while it remained stable in the mutants (Fig. 6C).

The pattern was different for POD (Fig. 6D), GR (Fig. 6E), and GST (Fig. 6F). Here, the activities of these enzymes were elevated in the mutants in response to salt stress, whereas the wild type did not show significant changes. In summary, it was found that the antioxidative enzyme machinery in *cpm2* and *hebiba* is altered compared with the wild type, whereby the activities of APX and SOD in response to salt were down-regulated, while the activities of POD and GST were up-regulated in the mutants. Mild differences in the activity of several enzymes were also detected in the untreated control plants, indicating that JA affects the antioxidative activity not only in response to stress but also during normal development of plants.

### JA deficiency alters gene expression in response to salt stress

**Fig. 7.**
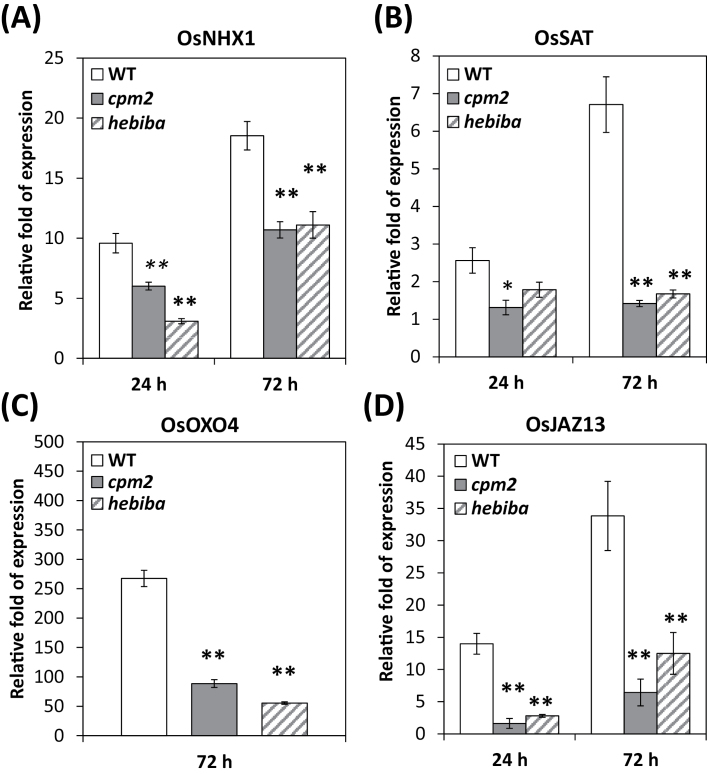
Alterations in transcript accumulations of stress-related genes in response to salinity. Plants were treated with 100mM NaCl for 24h and 72h as indicated. Values represent the mean of at least three independent experiments ±SE. Results for the wild type (WT), *cpm2*, and *hebiba* are indicated by white, grey, and striped bars, respectively. *Significant difference at *P*<0.05; **significant difference at *P*<0.01 in a Student’s *t*-testt.

In order to assess the adaptation of rice plants to salinity, the expression of salt-stress-related genes was examined after 24h and 72h of growth on saline medium. In both the mutants and the wild type, the relative expression of the *OsNHX1* gene (encoding a vacuolar Na^+^/H^+^ vacuolar antiporter) was induced by salinity (Fig. 7). However, levels were reduced by 40% in the mutants as compared with the wild type at the two time points investigated. A similarly different pattern was also detected for a gene encoding a protein similar to the enzyme serine acetyltransferase important for oxidative stress responses in *Arabidopsis* (Dominguez-Solis *et al.*, 2008), referred to as *OsSAT* (AK287779). In contrast to *OsNHX1*, this gene was only slightly induced after 24h, but showed an almost 7-fold increase in its transcripts at 72h. However, this gene was not induced in *cpm2* and *hebiba*. Expression of *OsOXO.4* (encoding oxalic acid oxidase, catalysing the production of H_2_O_2_ and CO_2_ from oxalic acid) was strongly induced under salinity stress (Fig. 7), but again its induction in the mutants was only 30% (*cpm2*) or even 20% (*hebiba*) of that observed in the wild type.

In order to investigate the role of JA signalling in modulating the salinity stress response, for the transcript abundance of *OsJAZ13* (encoding JAZ protein 13) was determined as a marker of JA signalling. The expression of *OsJAZ13* was induced by salt stress. However, the relative change of transcripts was found to be significantly smaller in the mutants at both time points examined (Fig. 7). Nevertheless, the JA biosynthesis mutants showed some induction of this gene, although this induction was only marginal as compared with the wild type.

While *OsOXO.4* and *OsJAZ13* transcripts accumulated strongly under salinity stress, other typical JA-responsive genes did not show such clear relative changes. The relative change of the transcripts of *OsJAR1* and *OsJAZ8*, two JA-dependent genes which are inducible by wounding, was weak and occurred late (Supplementary Fig. S5 at *JXB* online), suggesting that an only a subset of JA-dependent genes responds to salt stress.

### OPDA accumulates in response to salt stress

**Fig. 8.**
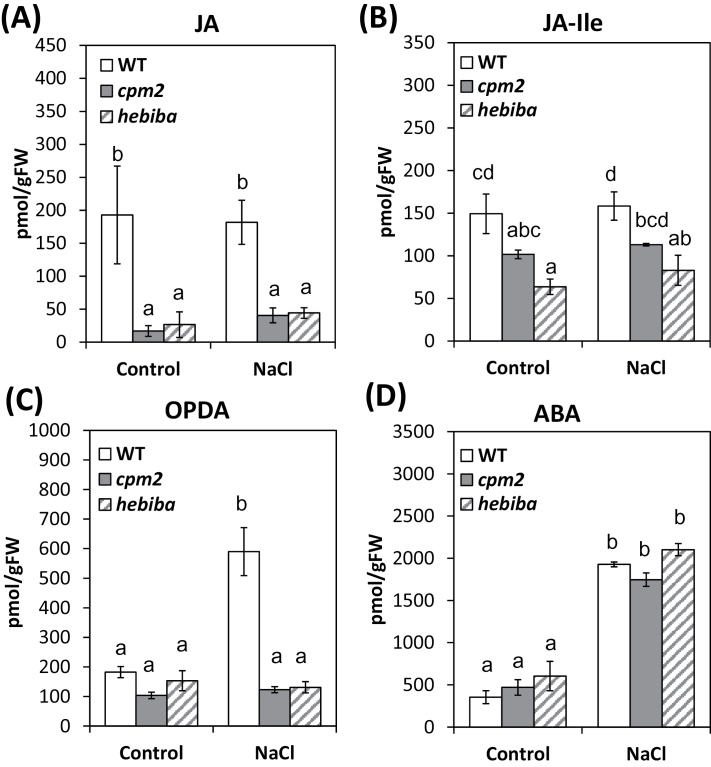
The level of jasmonates in shoots of the wild type (WT) and JA biosynthesis mutants under control conditions and 6h of salinity stress. Levels of (A) jasmonic acid (JA), (B) JA–isoleucine (JA-Ile), (C) 12-*cis*-oxophytodienoic acid (OPDA), and (D) abscisic acid (ABA). Values represent the mean of at least three independent experiments ±SE. Results for the WT, *cpm2*, and *hebiba* are indicated by white, grey, and striped bars, respectively. Means followed by different letters among treatments are significantly different, according to ANOVA single-factor test with respect to Tukey’s Honestly Significant Difference (HSD) test (*P*<0.05).

Levels of OPDA, JA, JA-Ile, and ABA in response to salt stress were compared in the wild type, *cpm2*, and *hebiba*. Since the early response was of interest, hormonal levels were compared 6h after challenging the plants with NaCl. As expected, the mutants produced less JA and JA-Ile compared with the wild type (Fig. 8A, B), but no significant increase in JA or JA-Ile levels in response to salt stress was observed, either in the wild type or in either of the mutants. However, in the wild type, OPDA levels increased ~3-fold in response to NaCl, while in the mutants OPDA levels remained very low even after salt stress (Fig. 8C).

Because the absence of JAs in the mutants may affect ABA biosynthesis, ABA levels were also examined in the same samples. This hormone was clearly induced (~4-fold) by NaCl treatment, but no significant differences in the levels were observed between the different genotypes.

## Discussion

The function of JA in salt stress is under debate. On the one hand, exogenous application of MeJA leads to the reduction of sodium uptake in salt-sensitive, but not in salt-tolerant rice cultivars (Kang *et al.*, 2005). On the other hand, exogenous application of MeJA promoted the translocation of sodium ions from root to shoot in maize, but at the same time reduced the overall amount of sodium ions in both roots and shoots (Shahzad, 2011). Part of the discrepancies in the literature might be caused by the fact that the constitutive presence of exogenous MeJA is not equivalent to the tightly controlled and mostly transient accumulation of endogenous JAs observed in the context of stress adaptation (for a review, see Ismail *et al.*, 2014*b*). To address the function of endogenous jasmonates, the strategy of analysing the salinity responses of jasmonate synthesis rice mutants was therefore employed. Surprisingly, these mutants performed significantly better than the wild type if challenged by salt stress. They showed fewer salt damage symptoms with respect to wilting of the second and third leaf (Fig. 1), produced longer roots under medium and high concentrations of NaCl (Fig. 2), and maintained a higher content of chlorophyll under salt stress (Supplementary Fig. S1 at *JXB* online). A detailed analysis of the mutant response revealed three potential phenomena that correlated with this improved salt tolerance: (i) a reduced translocation of sodium into the shoots; (ii) an elevated induction of antioxidants; and (iii) a lack of salt induction of the JA precursor OPDA. In the following, the potential role of these phenomena in salt tolerance or adaptation is discussed.

### Jasmonate synthesis mutants translocate less sodium into the shoots

The mutants accumulated smaller amounts of sodium ions in the leaves (Fig. 3). However, the concentration of sodium in the roots was generally much lower than that observed in the leaves, and here the mutants and wild type displayed the same values. The low concentration of sodium in the roots is consistent with the assumption that these concentrations reflect the steady-state levels resulting from uptake, translocation into the leaves, and extrusion (for a review, see Munns and Tester, 2008), whereas the leaves act as sodium sinks. However, it should further be kept in mind that the sodium measurements cannot discriminate between intracellular and apoplastic sodium in the root. The fact that the dose–response curve of root growth as regards sodium is shifted to higher concentrations in both *cpm2* and *hebiba* (Fig. 2) indicates that the effective concentration inside the cells of the root might well be lower in the mutants. It should be noted in this context that the 30% reduction of sodium accumulation in the leaves matched closely the shift of the dose–response curves measured for root growth. Moreover, for the *cpm2* mutant, the somewhat weaker reduction of sodium in the leaves is mirrored by a somewhat weaker shift of the dose–response curve for root growth as compared with *hebiba*. These correlations are consistent with a working model, where the reduced translocation of sodium into the symplasmic transport pathway (Erickson, 1986) accounts for the higher salt tolerance of the mutants. The present data are consistent with the published record on salt-tolerant varieties of rice. For instance, one of the most important parameters that distinguishes the rice salt-tolerant cultivars, such as Pokkali, from salt-sensitive cultivars, such as IR29, is their ability to take up less sodium ions into the leaves when exposed to salt stress (Golldack *et al.*, 2003; Kader and Lindberg, 2005). When, in the mutants, less sodium is translocated to the leaves, but the steady-state level of sodium in the roots does not change, this means either that uptake of sodium through the non-selective cation channels (Ismail *et al.*, 2014*a*) is reduced, and/or that the extrusion of sodium through the SOS1 exporter is promoted (for a review, see Munns and Tester, 2008). Irrespective of the underlying mechanism, the elevated salt tolerance of the JA biosynthesis mutants correlates with a reduced translocation of sodium ions into the photosynthetic tissues.

### Jasmonate synthesis mutants show elevated induction of antioxidants

ROS accompany many abiotic and biotic stress responses and, although often just considered as a manifestation of stress-related cellular damage, they play a second role as important signals triggering stress adaptation (for a review, see Dat *et al.*, 2000). To use a cellular event as a signal requires that the cell can control both its generation and its elimination. The wild type and mutants were therefore probed for indications of oxidative damage (Pang and Wang, 2008), and for indications of active control of ROS by salt-inducible antioxidants. This set of experiments supports two conclusions: (i) JA biosynthesis mutants display fewer indications of oxidative stress upon challenge by salinity and (ii) JA biosynthesis mutants induce antioxidants more readily upon challenge by salinity stress.

As a first readout for oxidative stress, MDA, an important intermediate in ROS scavenging widely used as an indicator of the extent of oxidation damage under stress (Borsani *et al.*, 2001; Apel and Hirt, 2004), was measured. The MDA level was estimated under salinity, and it was found that its level was significantly lower in the leaves of mutants compared with the wild type exactly parallel with the reduced translocation of sodium into the leaves (compare Figs 4A and 3). This parallelism is to be expected—when less salt is transferred into the leaves this should evoke less oxidative damage in the leaves. A similar result was reported by Vaidyanathan *et al.* (2003), where the MDA level was lower in Pokkali (a salt-tolerant rice cultivar) compared with Pusa Basmati (a salt-sensitive rice cultivar).

As a second readout for oxidative stress, the level of H_2_O_2_ was measured (Fig. 4B). H_2_O_2_ is not a free radical (Halliwell *et al.*, 2000), and is therefore comparatively innocuous. However, if converted non-enzymatically, the extremely noxious hydroxyl radical anion (OH·) will be generated which reacts strongly and rapidly (in <1 μs) with proteins, lipids, and DNA, causing cell membrane damage (Ishada *et al.*, 1999). Accumulation of H_2_O_2_ in the context of salinity damage has been documented for rice and soybean (Mandhania *et al.*, 2006; Weisany *et al.*, 2012). Similarly to MDA, the mutants do not show elevated levels of H_2_O_2_ under salinity, consistent with the reduced translocation of sodium into the leaves (compare Figs 4B and 3). In accordance with the observations described, two genes induced by oxidative stress (*OsOXO.4* and *OsSAT*) were not induced as strongly in the JA biosynthesis mutants as in the wild type (Fig. 7).

As a third readout for oxidative stress, soluble proline was measured. Soluble proline is considered as an osmoprotectant, probably associated with osmotic regulation and membrane stability under stress (Maggio *et al.*, 2000). Although its role under salt stress is not well established, there are many reports, for example supporting a function for proline in osmotic adjustment under salt stress in rice and wheat (Lutts *et al.*, 1996; Poustini *et al.*, 2007). On the other hand, accumulation of proline seems to accompany salinity-induced damage in rice (Garcia *et al.*, 1997; Hoai *et al.*, 2003). In fact, similarly to MDA and H_2_O_2_, increased levels of soluble proline were observed under salt stress. However, these increases were significantly lower in the mutants. Again this can be explained by the lower translocation of sodium ions into the mutant leaves. Proline accumulation is therefore seen as an indicator for salinity damage rather than as an indicator for stress tolerance.

Plants are equipped with an array of enzymatic and non-enzymatic antioxidative molecules to alleviate cellular damage caused by ROS (Foyer and Noctor, 2000). Whereas the reduced salinity damage in mutant leaves can be understood mostly in terms of reduced sodium translocation into the transpiration stream, there might also be differences in the capacity or inducibility of the antioxidative system. Among the enzymatic antioxidants, the activity of APX was strongly induced in the wild type, but not in the mutants. This enzyme catalyses the reduction of H_2_O_2_ to water and oxygen using ascorbate as an electron donor (Asada, 1999) and, since the mutants accumulate less H_2_O_2_ under salt stress (Fig. 4B), their need to rely on APX is less pronounced. Again, this can be explained in terms of reduced sodium translocation and would classify the induction of APX activity as a direct response to salinity-related damage.

However, not all antioxidant responses fell into this pattern. Some events showed an inverse pattern: for instance, the non-enzymatic antioxidative activity measured by the DPPH assay was higher for leaf extracts from challenged JA biosynthesis mutants as compared with the wild type. Moreover, the activities of POD, GR, and especially GST were induced by salt in the mutants, but not in the wild type. When a phenomenon is induced more strongly, although sodium levels are lower, this means that the respective event cannot be a mere manifestation of salinity-induced damage, but probably is adaptive in nature. Peroxidases comprise a group of specific enzymes such as NADPH-peroxidase, NADP-peroxidase, fatty-acid peroxidase, and others, and a group of non-specific enzymes from different sources catalyse the dehydrogenation of antioxidants such as phenolics, aromatic amines, and others in order to break down and/or produce H_2_O_2_ and ROS (Malik and Singh, 1980; Donald and Cipollini, 1997). The salt-induced higher POD activity in the mutants might be linked to a better scavenging activity for H_2_O_2_ which could be one reason for the lower H_2_O_2_ level in the salt-stressed mutants (Fig. 6D). GR and GST are well known to confer general protection against various stress conditions by detoxifying the cellular products of oxidative stress (Marrs, 1996; Yousof *et al.*, 2012). The stronger induction of non-enzymatic antioxidants, as well as the enzymatic activities of POD, GR, and GST in the mutants is observed on the basis of a significantly reduced translocation of sodium, and therefore on the basis of a significantly reduced level of sodium-induced damage. This stronger induction must therefore be caused by elevated sensitivity of sodium-triggered signalling and is evidence for an improved stress adaptation of the JA biosynthesis mutants.

### The overlooked damage signal: OPDA

The most striking observation of the plant hormone analysis was a strong increase of OPDA in the wild type by salt stress (Fig. 8). It is known that OPDA, an intermediate of JA biosynthesis, can by itself induce a specific set of genes which are not regulated by JA, and therefore OPDA can be regarded as a signalling molecule in its own right (Taki *et al.*, 2005). OPDA is also discussed as one of the highly reactive electrophile species (RES) responsible for signalling in chloroplasts (for a review, see Farmer and Müller, 2013). Upon binding to its receptor cyclophilin 20–3, OPDA can induce retrograde signalling (Park *et al.*, 2013). When the receptor-–hormone complex interacts with serine acetyltransferase, which stabilizes the formation of cysteine synthase, the redox homeostasis in plastids is altered. This signalling pathway is not active in the mutants, and this may be advantageous for the adaptation of rice to salt stress. In future, once suitable mutants in rice are identified, it will be interesting to compare *aoc* mutants and mutants of OPDA REDUCTASE, which are able to synthesize OPDA, but not other JAs, in order to compare their sensitivity to salt.

**Fig. 9.**
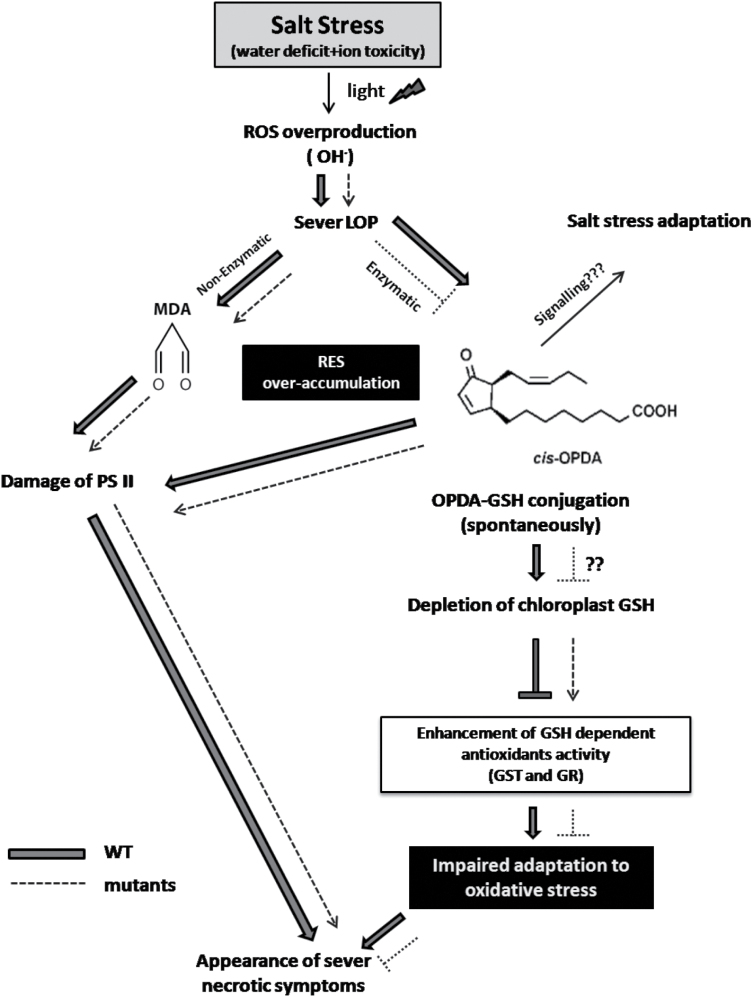
A model representing the effects of salt stress on the wild type and jasmonate biosynthesis mutants under salt stress. The observed phenotype of increased salt sensitivity in the wild type could be linked to the accumulation of higher amounts of MDA and OPDA as a result of higher lipid peroxidation due to ROS accumulation. Both MDA and OPDA are thought to destroy PSII and induce GSH depletion, resulting in disarmed antioxidative ability in the wild type. The mutants showed less accumulation of OPDA and MDA under salt stress conditions and less necrotic symptoms.

In the proposed model (Fig. 9), the accumulation of more sodium ions in the wild-type leaves in concert with the NaCl-triggered water deficit stress led to overproduction of ROS, especially OH· radicals. This will cause vigorous lipid peroxidation (LOP) (Ishada *et al.*, 1999; Mittler *et al.*, 2004; Halliwell, 2006). LOP in turn will cause, through enzymatic (eLOP) and non-enzymatic (nLOP) routes, the accumulation of RES, such as MDA and OPDA (Farmer and Müller, 2013). Since eLOP is blocked in the JA biosynthesis mutants due to inactivation of AOC (Riemann *et al.*, 2013), OPDA levels will be much higher in the wild type than in *cpm2* and *hebiba* (Fig. 8C). As a result, the wild type will be depleted of free GSH (a nucleophilic thiol-containing antioxidant) as result of stronger spontaneous OPDA–GSH conjugation (Davione *et al.*, 2006). Additionally, the higher abundance of OPDA and MDA is expected to impair the electron transport system in photosystem II (PSII; Alméras *et al.*, 2003). The model predicts higher levels of free GSH, which should result in a higher activity of GSH-dependent antioxidant enzymes such as GST and GR (Chen *et al.*, 2012). Consistent with this prediction from this model, both mutants, in contrast to the wild type, showed elevated activities of GR and GST under salinity stress (Fig. 6E, F).

As one of the most salt-sensitive glycophytes, rice has very limited effective strategies for dealing with salt stress and does not grow well on saline soil (Galvan-Ampudia and Testerink, 2011). Therefore, lowering the amount of uploaded sodium into the transpiration stream is vital for adaptation under salt stress in rice seedlings. Since sodium contents in the roots were similar in roots and differed in the shoots (Fig. 3), it can be concluded that in the JA biosynthesis mutants less sodium is transferred from the roots to the shoots compared with the wild type. To explain this reduced transfer there are basically three mechanisms: (i) stimulation of stomatal closure resulting in reduction of the transpiration stream; (ii) stimulation of sodium extrusion in the roots by the SOS1 exporter; and (iii) reduction of sodium influx from the apoplastic transport route in the root cortex into the endodermal cells by reduced activity of non-selective cation channels (NSCCs). Future studies, combining molecular and cell biological approaches in a time-resolved manner, will be required to identify the underlying mechanism responsible for the reduced sodium translocation.

## Supplementary data

Supplementary data are available at *JXB* online.

Figure S1. The total chlorophyll contents of both WT and JA biosynthesis mutants under control and salt stress conditions.

Figure S2. Superoxide-scavenging assay (SOSA) of both wild-type and JA biosynthesis mutants under salinity stress.

Figure S3. Hydrogen peroxide-scavenging assay (HPSA) of both wild-type and JA biosynthesis mutants under salinity stress.

Figure S4. Hydroxyl radical-scavenging activity (HRSA) of both wild-type and JA biosynthesis mutants under salinity stress.

Figure S5. Alterations in transcript accumulations of stress-related genes in response to salinity.

Table S1. The sequences of forward and reverse primers for the genes of interest and the two genes used for normalization.

Supplementary Data
