# Supplementary material for: Increased tolerance to salt stress in OPDA-deficient rice ALLENE OXIDE CYCLASE mutants is linked to an increased ROS-scavenging activity
Source: J Exp Bot. 2015 Apr 6;66(11):3339–52. doi: 10.1093/jxb/erv142 (PMC4449546; doi:10.1093/jxb/erv142)
Supplement: Supplementary Data [file supp_66_11_3339__index.html]

Increased tolerance to salt stress in OPDA-deficient rice ALLENE OXIDE CYCLASE mutants is linked to an increased ROS-scavenging activity — Increased tolerance to salt stress in OPDA-deficient rice ALLENE OXIDE CYCLASE mutants is linked to an increased ROS-scavenging activity — Supplementary Data 

# Increased tolerance to salt stress in OPDA-deficient rice *ALLENE OXIDE CYCLASE* mutants is linked to an increased ROS-scavenging activity

## Supplementary Data

Data files

**Files in this Data Supplement:**

- Supplementary Data - Supplementary Data
