# Supplementary material for: Increased tolerance to salt stress in OPDA-deficient rice ALLENE OXIDE CYCLASE mutants is linked to an increased ROS-scavenging activity
Source: J Exp Bot. 2015 Apr 6;66(11):3339–52. doi: 10.1093/jxb/erv142 (PMC4449546; doi:10.1093/jxb/erv142)
Supplement: Supplementary Data [file supp_erv142_jexbot143636_file001.pdf]

Journal of Experimental Botany, Increased tolerance to salt stress in OPDA-deficient rice *ALLENE OXIDE CYCLASE* mutants is linked with an increased ROS-scavenging activity

Mohamed Hazman, Bettina Hause, Elisabeth Eiche, Peter Nick, Michael Riemann

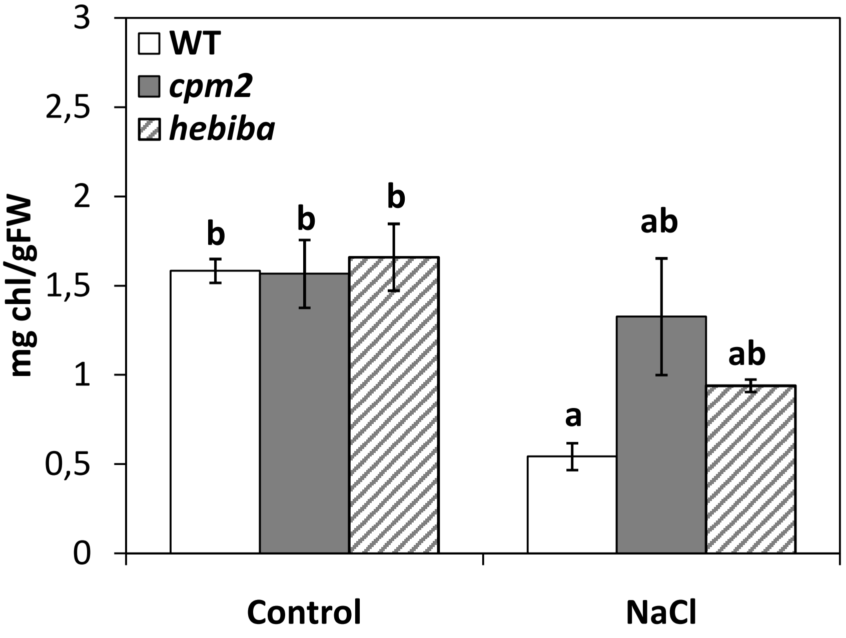

Supplementary Figure S1. The total chlorophyll contents of both WT and JA-biosynthesis mutants under control and salt stress conditions.

Values represent the mean of at least three independent experiments ±SE. Means followed by different letters among treatments are significantly different, according to ANOVA single factor test with respect to Tukey's Honest Significant Difference (HSD) test ($P<0.05$).

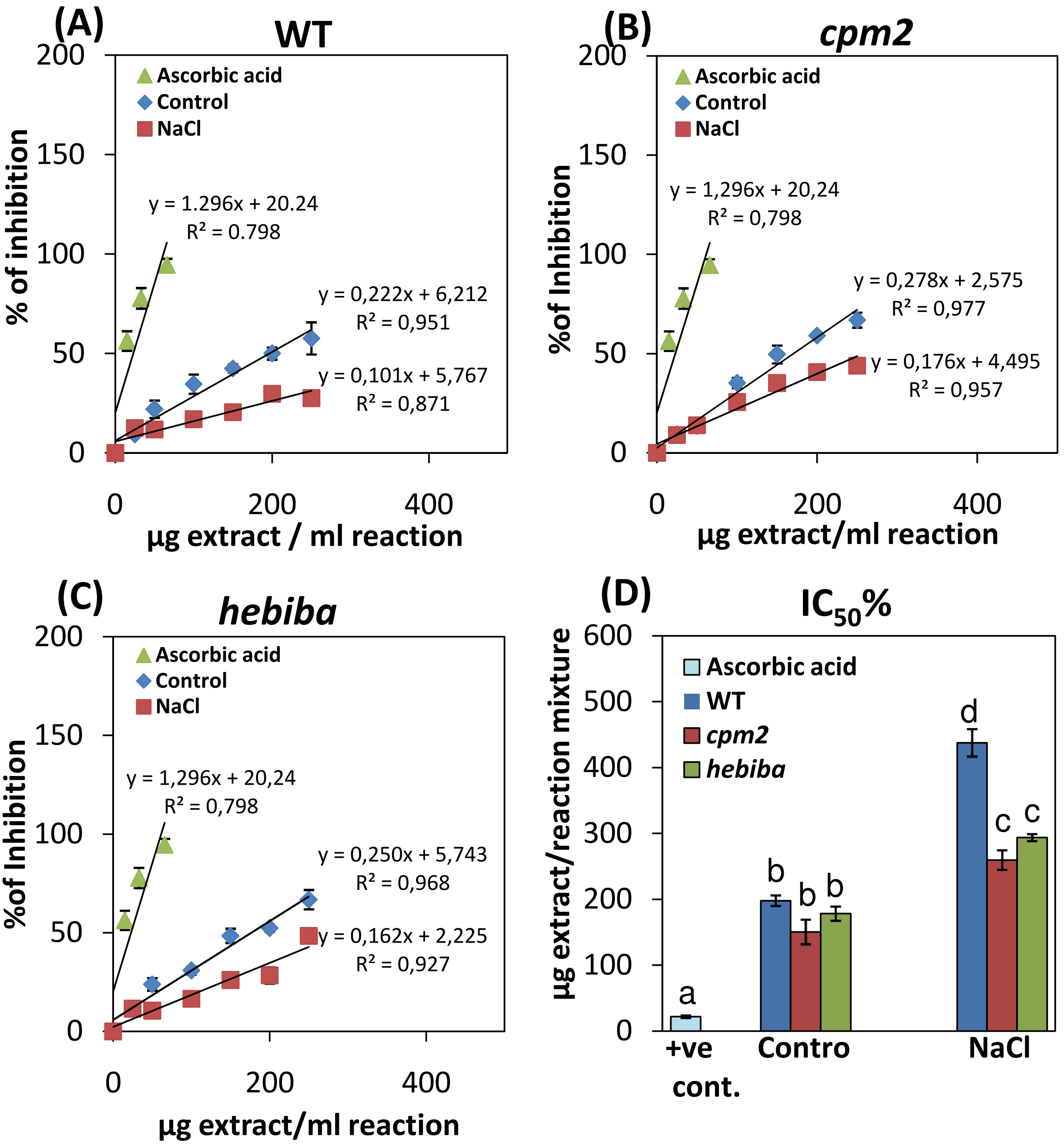

Supplemental Figure S2. Superoxide scavenging assay (SOSA) of both wild type and JA-biosynthesis mutants under salinity stress.

SOSA test of aqueous crude extracts of wild-type (A), *cpm2* (B) and *hebiba* (C) under control and salinity stress of 100 mM NaCl for 3 days. (D) IC50% values of WT, *cpm2* and *hebiba* under control (blank bars) and salt stress (grey bars) conditions, the standard ascorbic acid was used as internal positive control (Red bars). Values represent the mean of at least three independent experiments ±SE. Means followed by different letters among treatments are significantly different, according to ANOVA single factor test with respect to Tukey's Honest Significant Difference (HSD) test ($P<0.05$). Equations shown in the graph correspond to the trend lines of ascorbic acid, control and NaCl treatments from top to bottom, respectively.

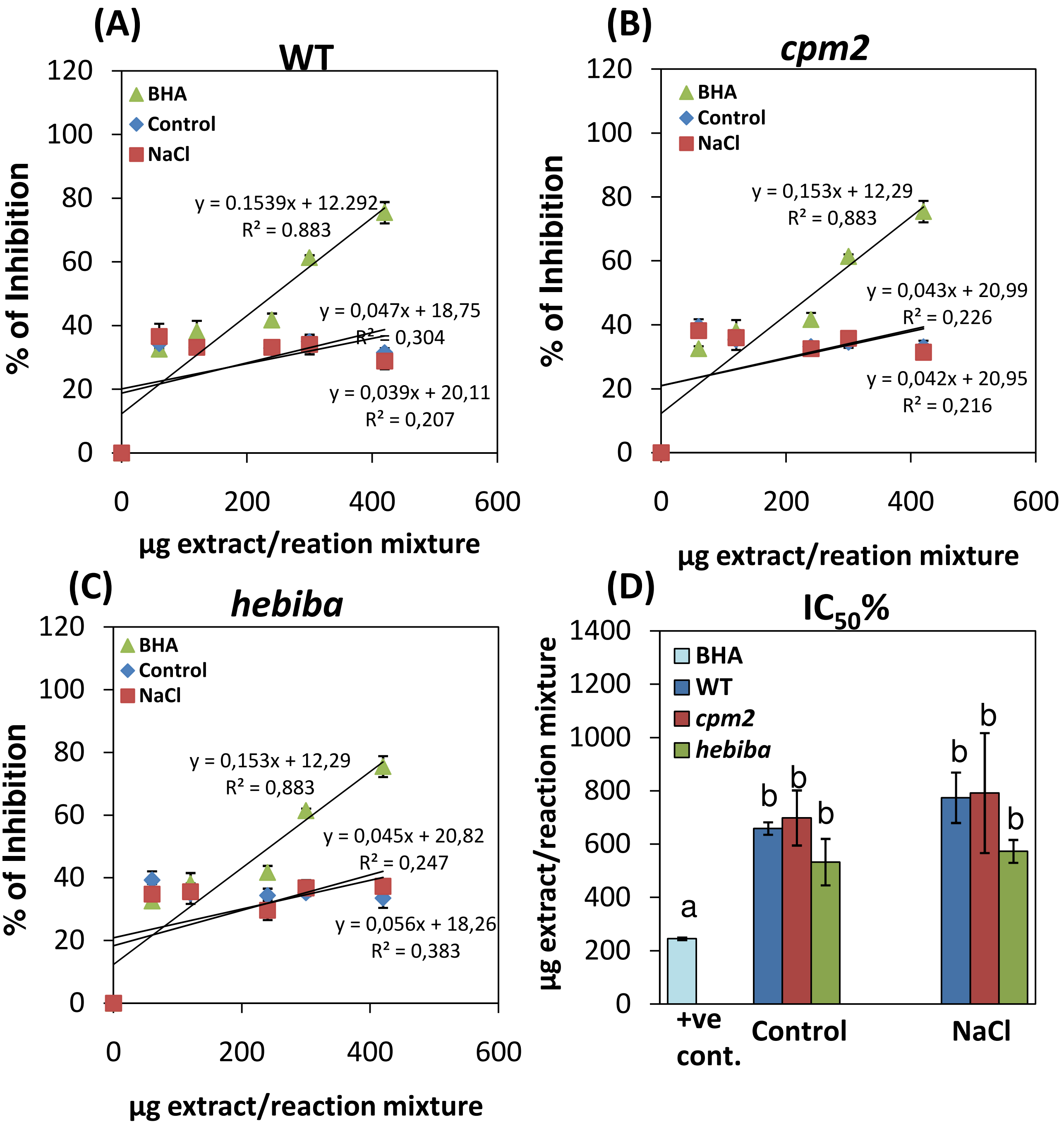

Supplemental Figure S3. Hydrogen peroxide scavenging assay (HPSA) of both wild type and JA-biosynthesis mutants under salinity stress.
HPSA test of aqueous extracts of wild-type (A), *cpm2* (B) and *hebiba* (C) under control and salinity stress of 100 mM NaCl for 3 days. (D) The values of IC50% of WT, *cpm2* and *hebiba* under control (blank bars) and salt stress conditions (grey bars), the standard BHA (butylated hydroxyanisole) (Red bars) was used as internal positive control. Values represent the mean of at least three independent experiments ±SE. Means followed by different letters among treatments are significantly different, according to ANOVA single factor test with respect to Tukey's Honest Significant Difference (HSD) test ($P<0.05$). Equations shown in the graph correspond to the trend lines of BHA, control and NaCl treatments from top to bottom, respectively.

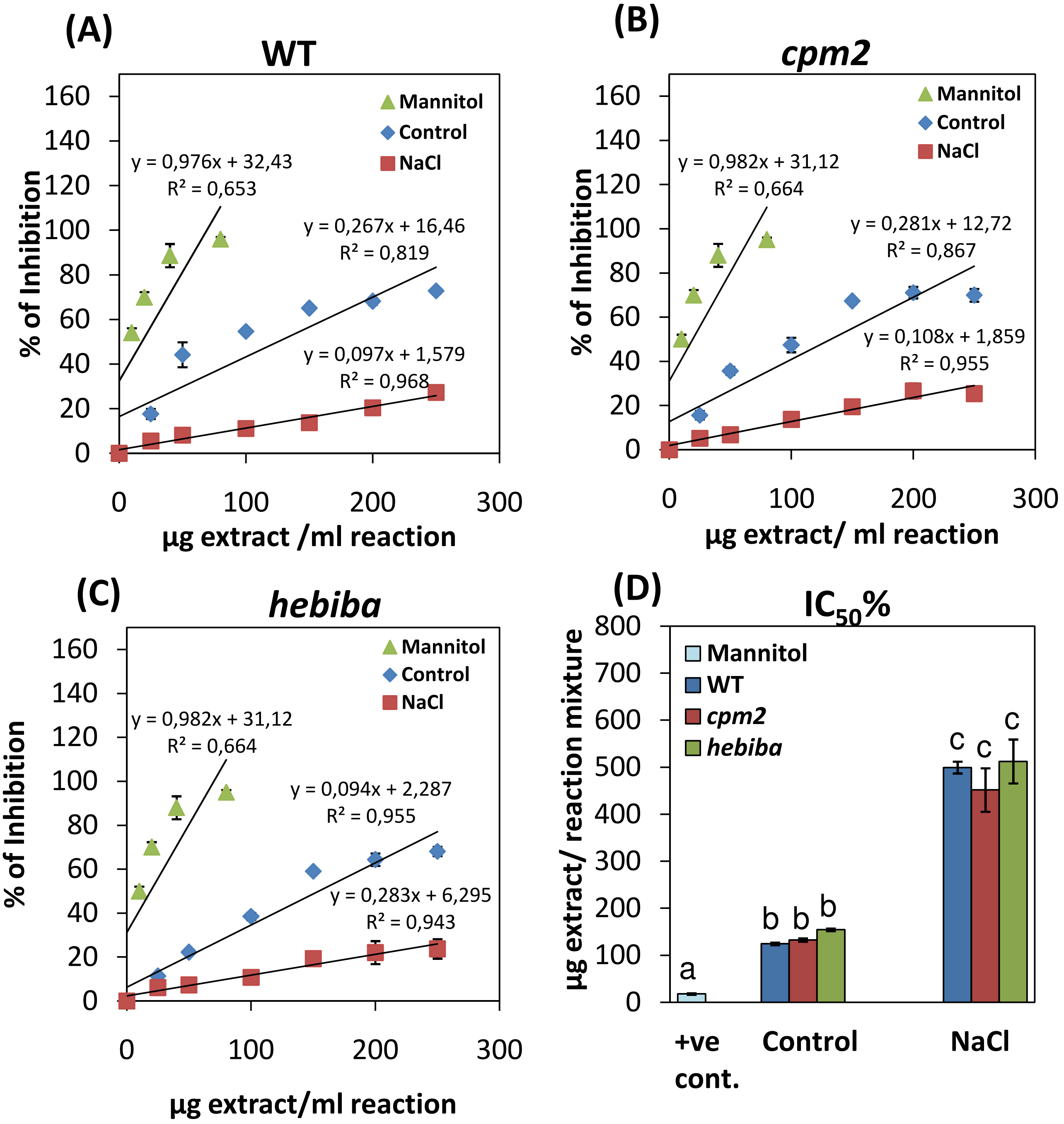

Supplemental Figure S4. Hydroxyl radical scavenging activity (HRSA) of both wild type and JA-biosynthesis mutants under salinity stress.

HRSA test of aqueous extracts of wild-type (A), *cpm2* (B) and *hebiba* (C) under control and salinity stress of 100 mM NaCl for 3 days. (D) The values of IC50% of WT, *cpm2* and *hebiba* under control (blank bars) and salt stress (grey bars) conditions, the standard mannitol was used as internal positive control (green bas). Values represent the mean of at least three independent experiments ± SE. Means followed by different letters among treatments are significantly different, according to ANOVA single factor test with respect to Tukey's Honest Significant Difference (HSD) test ($P<0.05$). Equations shown in the graph correspond to the trend lines of mannitol, control and NaCl treatments from top to bottom, respectively.

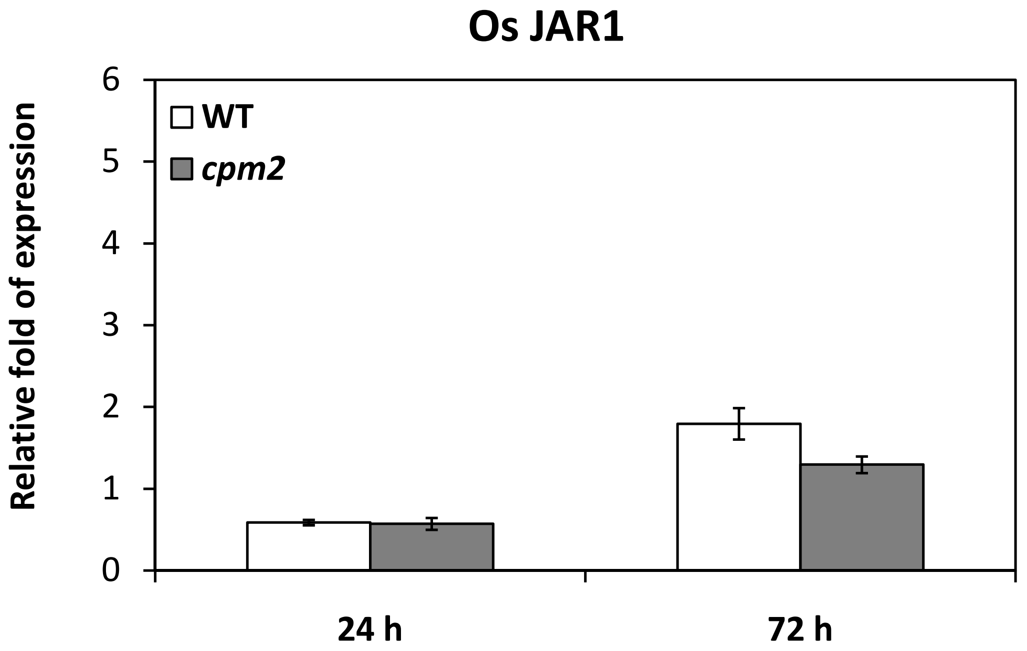

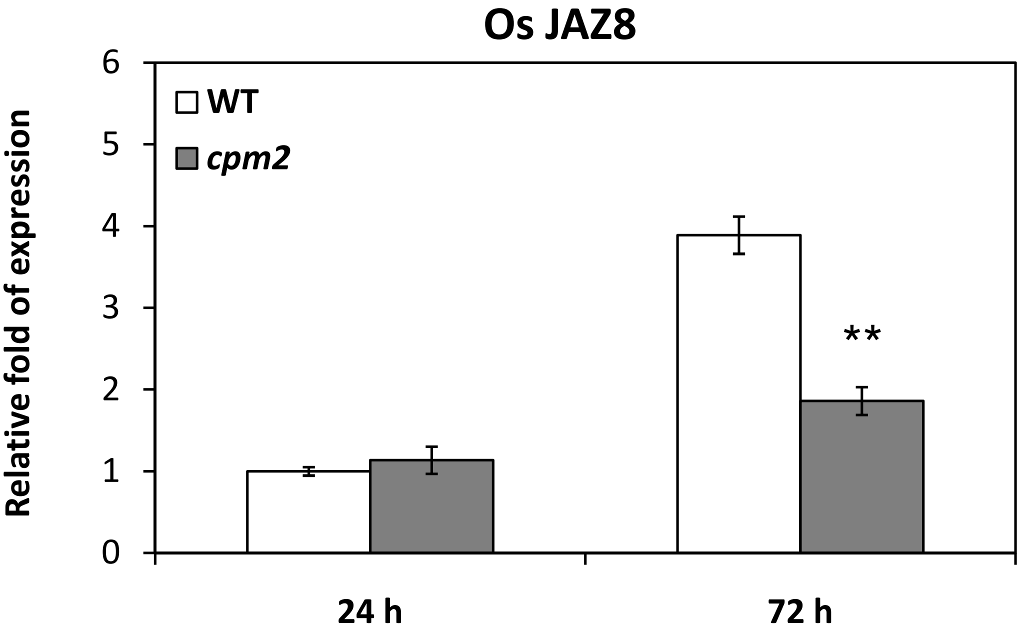

Supplementary Figure S5. Alterations in transcript accumulations of stress related genes in response to salinity.

Plants were treated with 100 mM NaCl for 24 h and 72 h as indicated, respectively. Results for the wild type and *cpm2* are indicated by white and grey bars (±SE), respectively. Two asterisks at P<0.01 in a Student's *t*-test.

Gene name	Accession No.	Forward(5'-3' prime)	Reverse(5'-3' prime)
Os NHX1	Os07t0666900	TGTGCTCCGACAACCTGTAA	TACATGCAGGGTGGCAACTA
Os SAT	Os05t0533500-01	TTGATTGGCAGGAAGAACG	TGGTGTAGTCCGACCACTGT
Os OXO.4	LOC_Os03g48750	AATAAACTTGTCGTCGTCGCCATC	GGCGCACTTACAAAATACC
Os JAZ13	Os10g25230	CGTGAGGATGCTTATTATGCTTG	CCAATGAAATTATATGATCCCTAGC
Os JAZ8	Os09g26780	GAAGGCTCAACAGCTGACCATAT	TTGGTGGACGGGAAGTTCTC
Os JAR1	Os05g50890	AGGAGGCATCAAAGTTCCTGGG	CTCAGCTCCCAGAAGATCACG
β-actin	AK101613	CCATTCTTCTCCGTCTT	GCTCCTGCTCGTAGTC
EF(TU)	Os02t0595700	CTTGATGCCACATGGAATTG	TTGTCAAGCAAGCAAACCAC

Supplementary Table S1. . The sequences of forward and reverse primers for the genes of interest and the two genes used for normalization.
